# Amyloidogenic, neuroinflammatory and memory dysfunction effects of HIV-1 gp120

**DOI:** 10.1007/s12272-021-01340-8

**Published:** 2021-07-23

**Authors:** Young-Jung Lee, In Jun Yeo, Dong Young Choi, Jaesuk Yun, Dong Ju Son, Sang-Bae Han, Jin Tae Hong

**Affiliations:** 1 College of Pharmacy and Medical Research Center, Chungbuk National University, Osongsaengmyeong 1-ro, Osong-eup, Heungdeok-gu, Cheongju, Chungbuk 28160 Republic of Korea; 2 College of Pharmacy, Yeungnam University, 280 Daehak Road, Gyeonsan, Gyeongbuk 38541 Republic of Korea; 3 Department of Equine Resources Science, School of Equine and Horticultural, Cheju Halla University, 38 Halladaehak-ro, Jeju-si, Jeju Special Self-Governing Province 63092 Republic of Korea

**Keywords:** HIV-1, gp120, Amyloid beta, Neuroinflammation

## Abstract

Human immunodeficiency virus 1 (HIV-1) infection can cause several HIV-associated neurocognitive disorders a variety of neurological impairments characterized by the loss of cortical and subcortical neurons and decreased cognitive and motor function. HIV-1 gp120, the major envelope glycoprotein on viral particles, acts as a binding protein for viral entry and is known to be an agent of neuronal cell death. To determine the mechanism of HIV-1 gp120-induced memory dysfunction, we performed mouse intracerebroventricular (i.c.v.) infusion with HIV-1 gp120 protein (300 ng per mouse) and investigated memory impairment and amyloidogenesis. Infusion of the HIV-1 gp120 protein induced memory dysfunction, which was evaluated using passive avoidance and water maze tests. Infusion of HIV-1 gp120 induced neuroinflammation, such as the release of iNOS and COX-2 and the activation of astrocytes and microglia and increased the mRNA and protein levels of IL-6, ICAM-1, M-CSF, TIM, and IL-2. In particular, we found that the infusion of HIV-1 gp120 induced the accumulation of amyloid plaques and signs of elevated amyloidogenesis, such as increased expression of amyloid precursor protein and BACE1 and increased β-secretase activity. Therefore, these studies suggest that HIV-1 gp120 may induce memory impairment through Aβ accumulation and neuroinflammation.

## Introduction

Alzheimer’s disease (AD) is the most common cause of dementia, accounting for 50–75% of all cases (Ferri et al. 2009; Lee et al. 2010) and the most common neurological complication. AD is defined by progressive synaptic impairment, excessive formation and accumulation of amyloid-beta (Aβ), and neuroinflammation (Blennow et al. 2006; Lee et al. 2010). Although the underlying mechanism of AD development remains unclear, experimental data have demonstrated that neuroinflammation-mediated Aβ accumulation in the brain may initiate and/or contribute to the process of AD development (Lee et al. 2010; Choi et al. 2012; Shadfar et al. 2015). It is well–known that viral proteins can activate macrophages, astrocytes and microglial cells that lead to the production of inflammatory molecules, further damaging neurons (Kaul et al. 2001).

Much of the associated research indicates that there are some common factors and pathways modulated in HIV^+^ and AD patients, thus suggesting some similarities exist between these two diseases. Among numerous factors, neuroinflammation is considered a common and crucial factor in the development and progression of two conditions. It has been reported that HIV-1 gp120, a fragment proteolytically cleaved from the Env protein, can activate neuroinflammatory pathways in the central nervous system (CNS), resulting in neuronal injury and dysfunction (Ortega and Ances 2014; Ru and Tang 2017). In addition, levels of cytokines, such as CCL3, IL-8, CCL2, IFN-γ, CXCL10, and IL-6, were found to be higher in HIV-1-infected individuals than in uninfected individuals (Kamat et al. 2012).

Furthermore, the envelope protein gp120 has been demonstrated to trigger the release of TNF-α and IL-1β which elicit neuronal apoptosis in numerous ex vivo and in vivo studies (Garden et al. 2002; Shah et al. 2011). Gp120-injected primary hippocampal cells have demonstrated the promotion of Aβ_1–42_ secretion (Aksenov et al. 2010). The brain of HIV^+^ patients showed increased expression of BACE1 (commonly observed with AD) (Stern et al. 2018). However, there are no definite data demonstrating a causal relationship between HIV-1 infection and neuronal damage or the onset of AD. Therefore, in the present study, we examined whether injection of the HIV-1 gp120 protein affects the development of AD and whether this effect is related to neuroinflammation-mediated Aβ-accumulation in the brain.

## Materials and methods

### HIV-1 gp120 infused mouse model

**Fig. 1:**
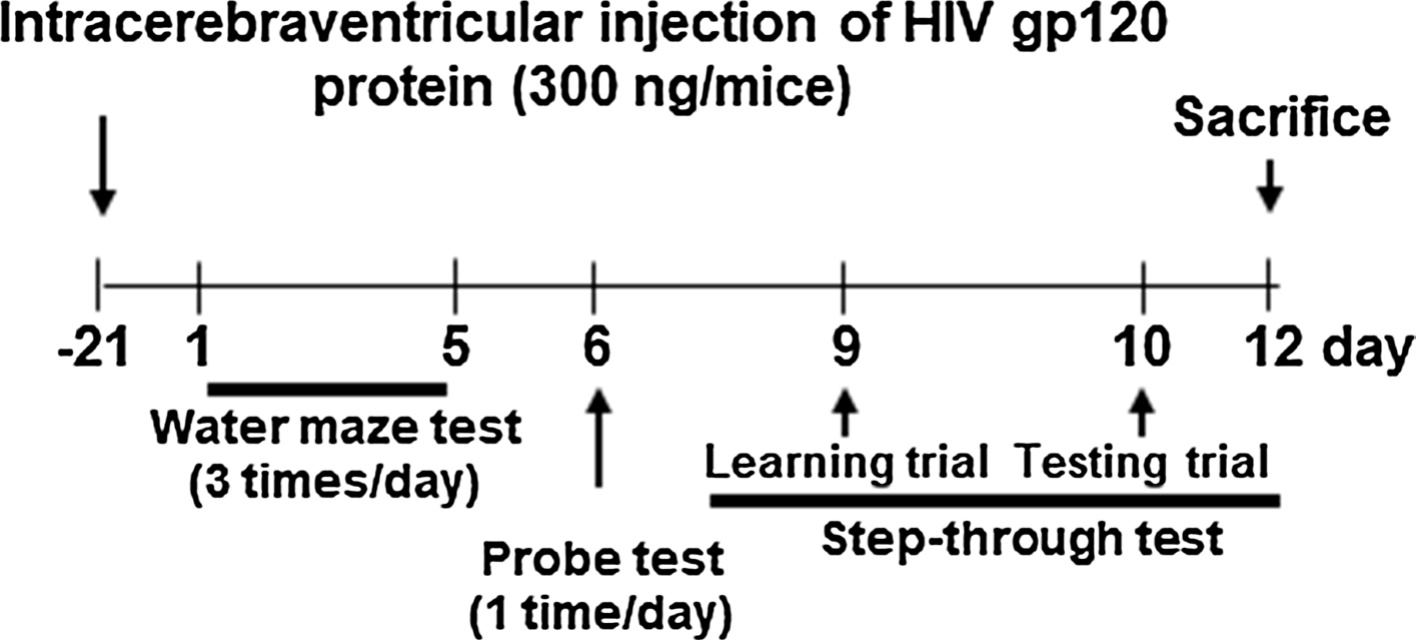
Experimental scheme. 300 ng gp120 was injected into the third ventricle of mice brain. After 21 days following injection, the memorial abnormalities were performed through behavioral tests of water maze test, probe test and step-through test, sequentially as shown in Fig. 1

A total of 20 5-week-old male ICR mice weighing 25–30 g (Samtako, Gyeonggi-do, Korea) were maintained in accordance with the Institutional Animal Care and Use Committee (IACUC) of Laboratory Animal Research Center at Chungbuk National University, Korea (CBNU-404-12-01). All mice were housed in a room that was automatically maintained at 21–25 °C and relative humidity (45–65%) with a controlled 12 h light–dark cycle. To study HIV-1 gp120-induced abnormalities, the mice were randomly allocated to two groups. Group 1 (control group; *n *= 10) was injected saline vehicles, and group 2 (HIV-1 gp120 group; *n *= 10) was injected HIV-1 gp120 protein. In each group, all administrations of only saline or saline containing HIV-1 gp120 protein were performed through intracerebroventricular (icv) injection. All mouse were anesthetized with ketamine/xylazine cocktail at 100 mg/kg ketamine and 10 mg/kg xylazine by injecting intraperitoneally (IP). The depth of anesthesia was monitored by the loss toe pinch reflex. The method of icv injection was summarized as below: 1 μl saline (0.9% NaCl) containing 300 ng gp120 was injected stereotaxically into the third ventricle to be 0.25 mm posterior to the bregma of mice 2.4 mm in depth (anteroposterior, − 0.25 mm; mediolateral, 0 mm; dorsal ventral, − 2.4 mm relative to the bregma). The next behavioral experiments were performed after 21 days following the injection. Recombinant HIV-1 IIIB Glycoprotein gp120 was purchased from ImmunoDX, LLC (Woburn, MA). Experimental scheme was shown in Fig. 1.

### Water maze test

The water maze test is also a widely accepted method for memory test, and we performed this test as described by Morris (1984). Maze testing was performed by the SMART-CS (Panlab, Barcelona, Spain) program and equipment. A circular plastic pool (height: 35 cm, diameter: 100 cm) was filled with non-toxic black ink water kept at 22–25 °C. An escape platform (height: 14.5 cm, diameter: 4.5 cm) was submerged 0.5–1 cm below the surface of the water in position. The test performed three times a day for 5 days during the acquisition phase (Days 1–5), with three randomized starting points. The position of the escape platform was kept constant. Each trial lasted for 60 s or ended as soon as the mice reached the submerged platform. Swimming pattern of each mouse was monitored and recorded by a camera mounted above the center of the pool, and the escape latency, escape distance and swimming speed were assessed by the SMART-LD program (Panlab, Barcelona, Spain). A quiet environment, constant water temperature was maintained throughout the experimental period. The water maze test was used in 10 mice per group.

### Probe test

A probe trial in order to assess memory consolidation was performed 24 h after the water maze tests. In this trial, the platform was removed from the tank, and the mice were allowed to swim freely. For these tests, percentage time in the target quadrant and target site crossings within 60 s was recorded. The time spent in the target quadrant is taken to indicate the degree of memory consolidation that has taken place after learning. Swimming pattern of each mouse was monitored by a camera above the center of the pool connected to a SMART-LD program described above. The probe test was used in 10 mice per group.

### Passive avoidance performance test

The passive avoidance test is also widely accepted as a simple and rapid test method for measuring memory capacity. The passive avoidance response was determined using a “step-through” apparatus (Med Associates, Inc., Georgia, VT, USA) that is consisted of an illuminated and a dark compartment (each 20.3 × 15.9 × 21.3 cm) adjoining each other through a small gate with a grid floor, 3.175 mm stainless steel rod set 8 mm apart. One day after water maze test, training trial was performed. The mice were placed in the illuminated compartment facing away from the dark compartment. When the mice moved completely into the dark compartment, it received an electric shock (1 mA, 3 s duration). Then the mice were returned to their home case. At 1 day later, the mice were placed in the illuminated compartment and the latency period to enter the dark compartment defined as “retention” was measured. The time when the mice entered in the dark compartment were recorded and described as step-through latency. The retention trials were set at a limit of 180 s of cut-off time. The passive avoidance performance test was used in 10 mice per group.

### Brain collection and preservation

After behavioral test (step through test), animals were perfused with PBS under inhaled ether anesthetization. The brains were immediately collected in the same manner and separated into cortical and hippocampal regions. All the brain regions were immediately stored at − 80 °C and used to for measure biological assay.

### Immunohistochemistry

Mice were anesthetized with ether. While under general anesthesia, the mice received intracardiac perfusion with 20 ml of saline. Brains were fixed in formalin and paraffin-enclosed for examination. 6 μm-thick tissue sections were used with immunohistochemistry. Paraffin-embedded sections were deparaffinized and rehydrated, washed in distilled water, and then subjected to heat-mediated antigen retrieval treatment. Endogenous peroxidase activity was quenched by incubation in 2% hydrogen peroxide in methanol for 30 min and then cleared in PBS for 5 min. The sections were blocked for 30 min with 3% normal horse serum diluted in PBS. Immunohistochemical staining was performed using the avidin–biotin–peroxidase method. The sections were incubated overnight at 4 °C with appropriate antibodies; Aβ_1–42_ (1:2000 dilution, Covance, Berlely, CA, USA), GFAP (1:5000, Abcam, Inc, Cambridge, MA, USA), Iba1 (1:5000, Wako, Osaka, Japan), iNOS (1:100, Abcam, Inc, Cambridge, MA, USA), COX-2 (1:100, Cayman Chemical, Ann Arbor, MI, USA) and BACE1 (1:500, Sigma St. Louis, MO, USA). After washing in PBS, the sections were incubated in biotinylated secondary antibodies (1:2000 dilution, Vector Laboratories, Burlingame, CA, USA) for 1 h at room temperature. The sections were subsequently washed and incubated with avidin-conjugated peroxidase complex (ABC kit, 1:200 dilution. Vector Laboratories) for 30 min followed by PBS washing. The peroxidase reaction was performed in PBS using 3,3′-diaminobenzidine tetrahydrochloride (DAB, 0.02%) as the chromogen. It was then counterstained by a hematoxylin. Finally, sections were dehydrated in ethanol, cleared in xylene, and mounted with Permount (Fisher Scientific, Hampton, NH), and evaluated on a light microscopy (Olympus, Tokyo, Japan). To determine the expression of iNOS, COX-2, BACE1, GFAP, and Iba1, the stained cells by each antibody were counted. The six sections with three different animal brains were analyzed, and cells at three randomly selected areas (100 × 100 μm) in each section were assessed. The immunoreactive cells by anti-iNOS, COX-2, BACE1, GFAP and Iba1 antibody were counted, and expressed as percentage of stained cells. The quantity of reactive cells was expressed as the average number of reactive cells per high power field (visible reactive cells/HPF).

To simultaneously determine level of Aβ, we performed immunofluorescence assay in paraffin section of brain. The sections were then incubated to mouse monoclonal antibody for Aβ_1–42_ (1:2000, Clone No. 4G8, Covance, Berkeley, CA, USA) overnight at 4 °C. After washes with ice-cold PBS, followed by treatment with an anti-mouse secondary antibody labeled with Alexa Flour 488 (1:100 dilution, Molecular Probes, Inc., Eugene, OR) for 2 h at room temperature, immunofluorescence images were acquired using a confocal laser scanning microscope (TCS SP2, Leica Microsystems AG, Wetzlar, Germany). Areas of amyloid deposition in mice brain were identified by staining of 0.2% thioflavine S (Sigma St. Louis, MO, USA) and microscopic evaluation. For detection of anti-Aβ antibody- and thioflavin S-double positive staining, digital images were acquired using a confocal laser scanning microscope (TCS SP2, Leica Microsystems AG, Werzlar, Germany).

### Western blotting analysis

Tissues were homogenized with lysis buffer [50 mM Tris pH 8.0, 150 mM sodium chloride (NaCl), 0.02% sodium azide, 0.2% sodium dodecyl sulfate (SDS), 1 mM phenylmethanesulphonyl fluoride, 10 μl/ml aprotinin, 1% IGAPEL CA-630, 10 mM sodium fluoride, 0.5 mM ethylenediaminetetraacetic acid (EDTA), 0.1 mM ethylene glycol tetraacetic acid and 0.5% sodium deoxycholate] and centrifuged at 15,000×*g* for 15 min. Equal amount of proteins (40 μg) were separated on a SDS/10 and 15% polyacrylamide gel and then transferred to a polyvinylidene difluoride membrane (GE Water and Process Technologies). Blots were blocked for 1 h at room temperature with 5% (w/v) non-fat dried milk in Tris-buffered saline [10 mM Tris (pH 8.0) and 150 mM NaCl] solution containing 0.05% Tween 20. The membrane was then incubated for 3 h at room temperature with specific antibodies: anti-C99, anti-APP (1:500, ABR-Affinity Bioreagents, Golden, CO, USA), anti-Aβ (1:500, 4G8, Covance, Berlely, CA, USA), anti-BACE1 (1:500, Sigma St. Louis, MO, USA), anti-β-actin (1:2000, Santa Cruz Biotechnology, Inc., Santa Cruz, CA, USA), anti-iNOS (1:100, Abcam, Inc, Cambridge, MA, USA), anti-COX-2 (1:100, Cayman Chemical, Ann Arbor, MI, USA), anti-GFAP (1:1000; Abcam Inc., Cambridge, MA, USA) and anti-Iba1 (1:1000; Abcam Inc., Cambridge, MA, USA) were used. The blots were then incubated with the corresponding peroxidase-conjugated anti-goat/rabbit/mouse antibodies (1:2000; Santa Cruz Biotechnology, Inc., Santa Cruz, CA, USA). Immunoreactive proteins were detected using ECL Western blotting detection system. The densitometric scanning of relative density of the protein bands was performed using MyImage (SLB, Seoul, South Korea) and quantified by Lab Works 4.0 (UVP Inc., Upland, CA, USA).

### Measurement of Aβ_1–42_

Lysates of brain tissue were obtained through protein extraction buffer containing protease inhibitor. Aβ_1–42_ levels were determined using specific ELISA Kit (Immuno-Biological Laboratories Co., Ltd., Takasaki-Shi, Gunma, Japan). In brief, 100 μl of sample was added into the pre-coated plate and was incubated for overnight at 4 °C. After washing each well of the precoated plate with washing buffer, 100 μl of labeled antibody solution was added and the mixture was incubated for 1 h at 4 °C in the dark. After washing, chromogen was added, and the mixture was incubated for 30 min at room temperature in the dark. Finally, the resulting color was assayed at 450 nm using a microplate absorbance reader (SunriseTM, TECAN, Switzerland) after adding stop solution.

### Measurement of β-secretase activity

β-secretase activity in the brains was determined using commercially available β-secretase fluorescence resonance energy transfer (BACE1 FRET) assay kit (PANVERA, Madison, USA) according to the manufacturer’s protocols and as described elsewhere. This formation of fluorescence was read using a Fluostar galaxy fluorometer (excitation at 355 nm and emission at 510 nm) with Felix software (BMG Labtechnologies). β-secretase activity was expressed as nmol/(mg protein-min).

### Reverse transcription PCR analysis

Total RNA was extracted using the RNAqueous kit (Applied Biosystems, Foster city, CA). The cDNA was synthesized using High-Capacity RNA-to-cDNA kit (Applied Biosystems, Foster city, CA) according to the manufacturer’s protocol. Briefly, 1 μg of total RNA was used for cDNA preparation. The primers for IL-16, soluble intercellular adhesion molecule-1 (ICAM-1), macrophage colony-stimulating factor (M-SCF), T cell immunoglobulin and mucin domain-1 (TIM-1), IL-2 and glyceraldehyde-3-phosphate dehydrogenase (GAPDH) as an internal PCR control were as follows: 5′-AAA TGG ACA CTG CCA ATG GTG CTC-3′ (sense) and 5′-AAA GGA GCT GAT TCT CTG CCG GAT-3′ (antisense) for IL-16, 5′-AAA CGG GAG ATG AAT GGT ACC TAC-3′ (sense) and 5′-TGC ACG TCC CTG GTG ATA CTC-3′ (antisense) for ICAM-1, 5′-AGT GGT CTG TAA GCT CCA TC-3′ (sense) and 5′-GAG CTT CTT GCA ATG GGT TG-3′ (antisense) for M-CSF, 5′-CTA TGT TGG CAT CTG CAT CG-3′ (sense) and 5′-AAG GCA ACC ACG CTT AGA GA-3′ (antisense) for TIM-1 and 5′-TCC CTC AAG ATT GTC AGC AA-3′ (sense) and 5′-AGA TCC ACA ACG GAT ACA TT-3′ (antisense) for GAPDH. All PCRs were run in a 7500 Real-Time PCR System (Applied Biosystems, Foster city, CA, USA). The PCR cycles consisted of denaturation at 94 °C for 30 s; annealing at 55 °C for 30 s (GAPDH), 50 °C for 30 s (M-CSF and ICAM-1) or 60 °C for 30 s (IL-16 and TIM-1); and extension at 72 °C for 90 s for 30 cycles. The PCR product was separated by electrophoresis on a 1.5% agarose gel, stained with ethidium bromide and then detected under UV light. The densitometric scanning of relative density of the PCR bands was quantified by Lab Works 4.0 (UVP Inc., Upland, CA, USA).

### Cytokine arrays

Mouse Cytokine Array Panels were used according to the manufacturer instructions (R&D Systems Inc., Minneapolis, MN, USA). Briefly, brain lysates were mixed with a cocktail of biotinylated detection antibodies prior to incubation at 4 °C with the array membranes. Following washing, streptavidin–horseradish peroxidase was applied for 30 min at ambient temperature. Immunoreactivity was then visualized using enhanced chemiluminescence reagent (GE Healthcare). Densitometric analysis was then performed using MyImage (SLB, Seoul, Korea) and quantified by Labworks 4.0 software (UVP Inc., Upland, CA, USA).

### Statistical analysis

The data were statistically analyzed using the GraphPad Prism software (version 4.03; GraphPad software, Inc., San Diego, CA, USA). Data are presented as mean ± SD. The group differences in all data were assessed by Student’s *t* test, one-way analysis of variance (ANOVA) followed by Bonferroni’s post hoc test. A value of *p* < 0.05 was considered statistically significant. # Significantly different between the saline-injected group and HIV-1 gp120-injected group (p < 0.05).

## Results

### HIV-1 gp120 induced learning and memory impairment through icv injection in mice

**Fig. 2:**
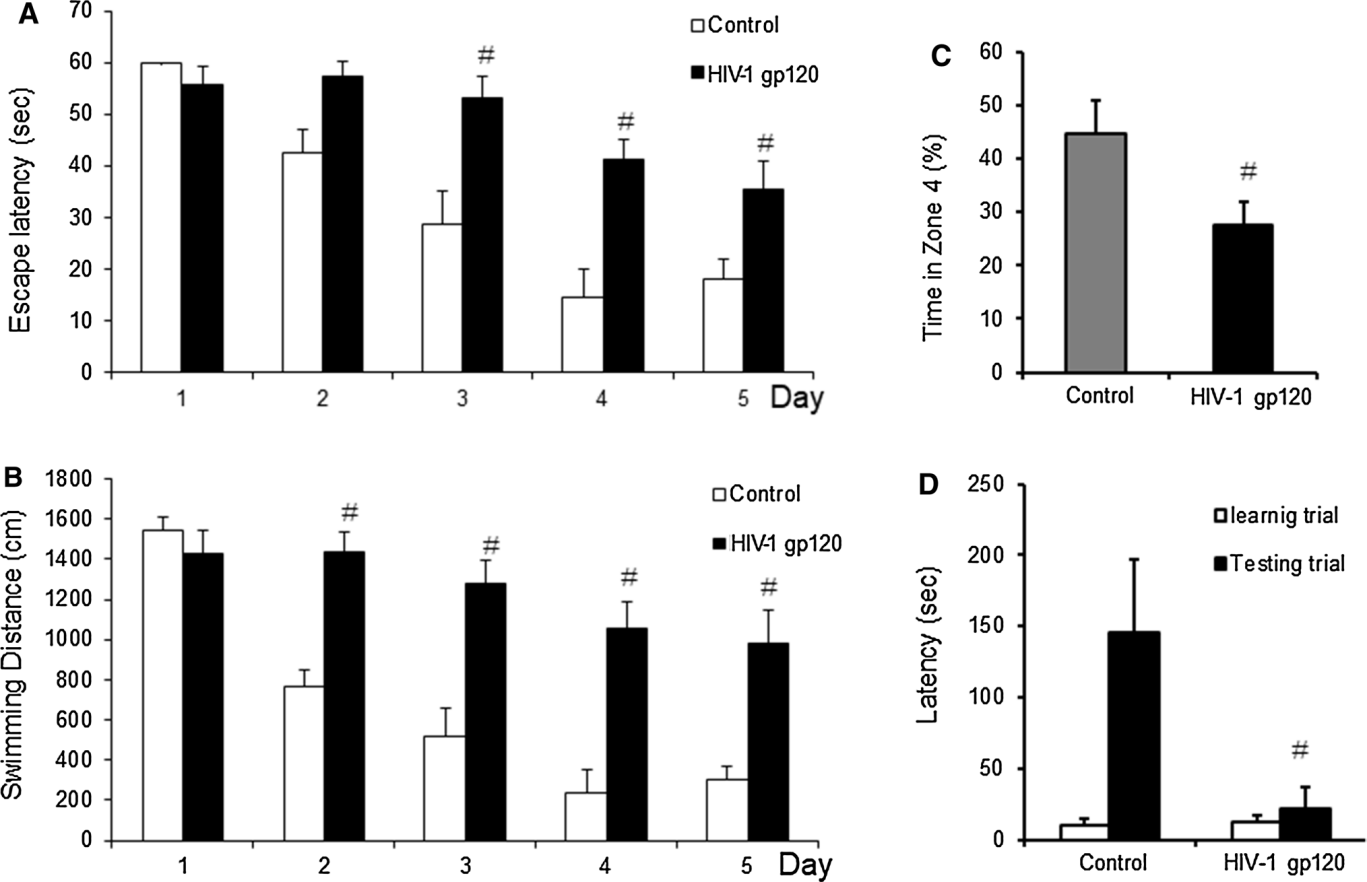
Memorial impaired effects on HIV-1 gp120-induced abnormalities. To investigate memorial impaired effects in HIV-1 gp120-induced abnormalities, we performed water maze test (**A**, **B**), probe test (**C**) and step-through type passive avoidance test (**D**). Memory function was determined by the escape latencies (**A**, s) and distance (**B**, cm) for 5 days, and time spent in target quadrant (**C**,  %) in probe test after HIV-1 gp120-injection. Each value is mean ± SD from 10 mice. #, Significantly different from control group (*p* < 0.05). Control, saline-injected group. HIV-1 gp120, HIV-1 gp120-injected group

To investigate HIV-1 gp120-induced memory impairment effects in HIV-1 gp120-injected mice, we injected HIV-1 gp120 into the third ventricle of mouse brains and compared the learning and memory abilities to these mice to saline-injected mice using water maze, probe, and step-through tests. The mice were trained with three trials per day over 5 days; we then analyzed their ability to locate and escape onto the platform, and their spatial learning scores were recorded. Significant differences between HIV-1 gp120-injected and control mice due to the memory impairment effect were seen on day two, and the impairment effect persisted for 5 days in the water maze test. The mice exhibited shorter escape latencies after training; however, the escape latency of the HIV-1 gp120-injected mice did not reduce as much as that of the saline-injected mice. HIV-1 gp120 in mice brains significantly impaired the memory ability of mice. Statistical analysis of data from days three to five showed the significance of the memory impairment effect of HIV-1 gp120 injection. The escape latency among the HIV-1 gp120-injected group (35.4 ± 5.6 s) on day five was greater than that of the saline-injected group (18.2 ± 3.7 s, *p *< 0.001, Fig. 2A). The swimming distance in the HIV-1 gp120-injected group (979.4 ± 167.9 cm) on day five was also longer than that of the saline-injected group (298.7 ± 67.5 cm, *p *< 0.001) (Fig. 2B). However, there was no significant difference in average speed between the saline-injected group and the HIV-1 gp120-injected group (data not shown). After the water maze test, we performed a probe test to assess the maintenance of memory. During the probe test, the time spent in the target quadrant by the HIV-1 gp120-injected group (27.6 ± 4.4%) was significantly decreased compared to that of the saline-injected group (44.7 ± 6.4%, *p *= 0.01, Fig. 2C). We also evaluated learning and memory capacities with a passive avoidance test using a step-through method. In the passive avoidance test learning trial, there was no significant difference between the two groups. However, in the test trial, the step-through latency of the saline-injected group (145.2 ± 51.8 s) had significantly decreased to 21.4 ± 15.8 s (*p *< 0.001) compared to the HIV-1 gp120-injection group (Fig. 2D).

### HIV-1 gp120 induced Aβ accumulation in mouse brain

**Fig. 3:**
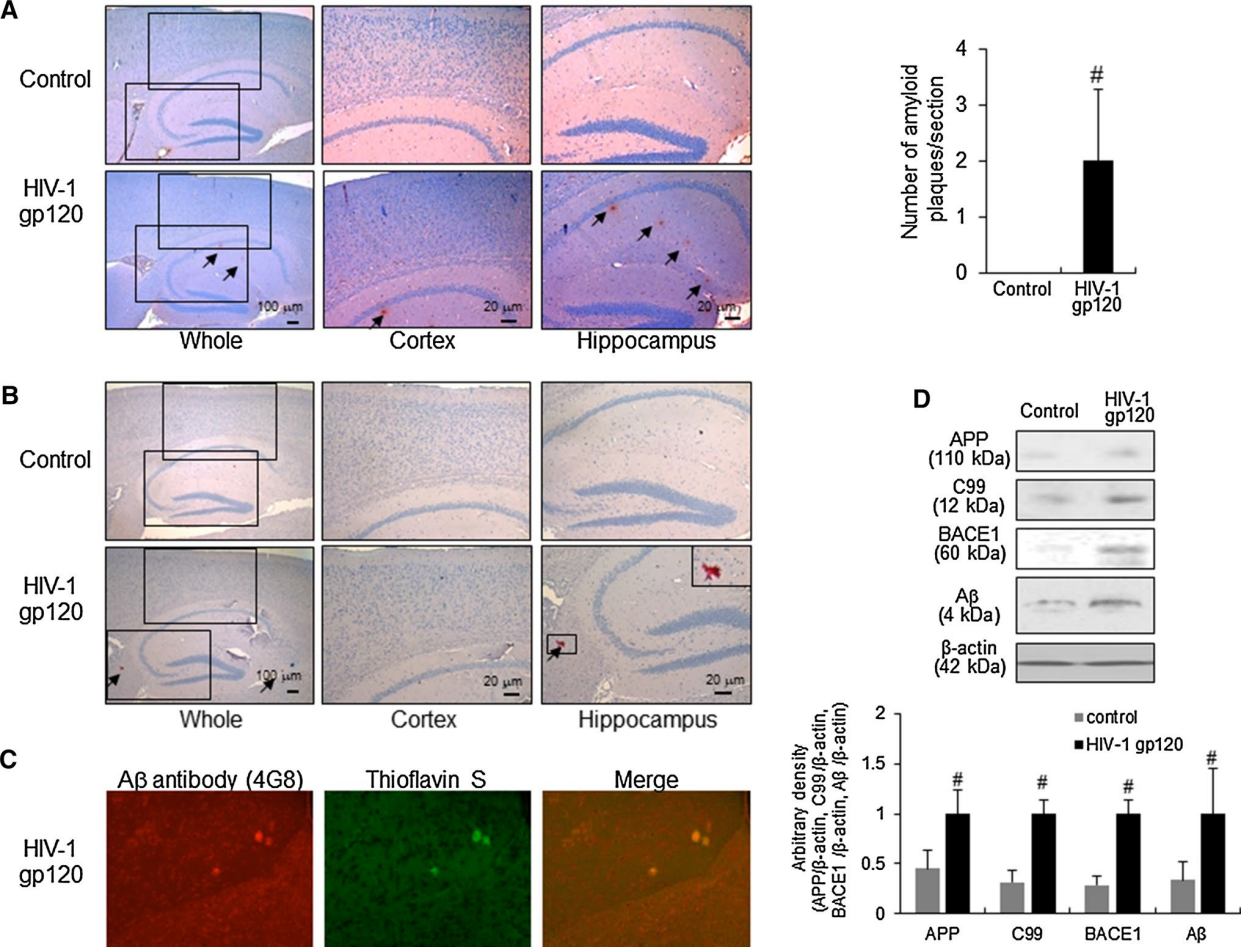
Aβ accumulation effect on HIV-1 gp120-injected mice brain. **A** Immunoreactive protein of anti-Aβ_1–42_ antibody was investigated in the cortex and hippocampus. 6 μm-thick sections of brains from mice were incubated with anti-Aβ_1–42_ antibody and counterstained with hematoxylin. Arrow indicates Aβ_1–42_ accumulation which is clearly higher in the cerebral cortex and hippocampus of HIV-1 gp120-injected mice. Amyloid plaque detection via congo red staining (**B**) and thioflavin S (**C**) was performed in the cortex and hippocampus. 6 μm-thick sections of brains were incubated with 0.2% congo red solution or thioflavin S solution for 20 min and counterstained with hematoxylin. Arrow indicates amyloid plaque which is clearly higher in the cerebral cortex and hippocampus of HIV-1 gp120-injected mice. **C** To define Aβ_1–42_ accumulation in HIV-1 gp120-injected mice brain, co-immunostaining using anti-Aβ_1–42_ antibody (red) and thioflavin S was performed. **D** The expression of APP, C99, Aβ_1–42_, BACE1 in mice brain were detected by western blotting using specific antibodies. β-Actin protein was used as an internal control. Each blot is representative for three experiments. Control, saline-injected group. HIV-1 gp120, HIV-1 gp120-injected group

**Fig. 4:**
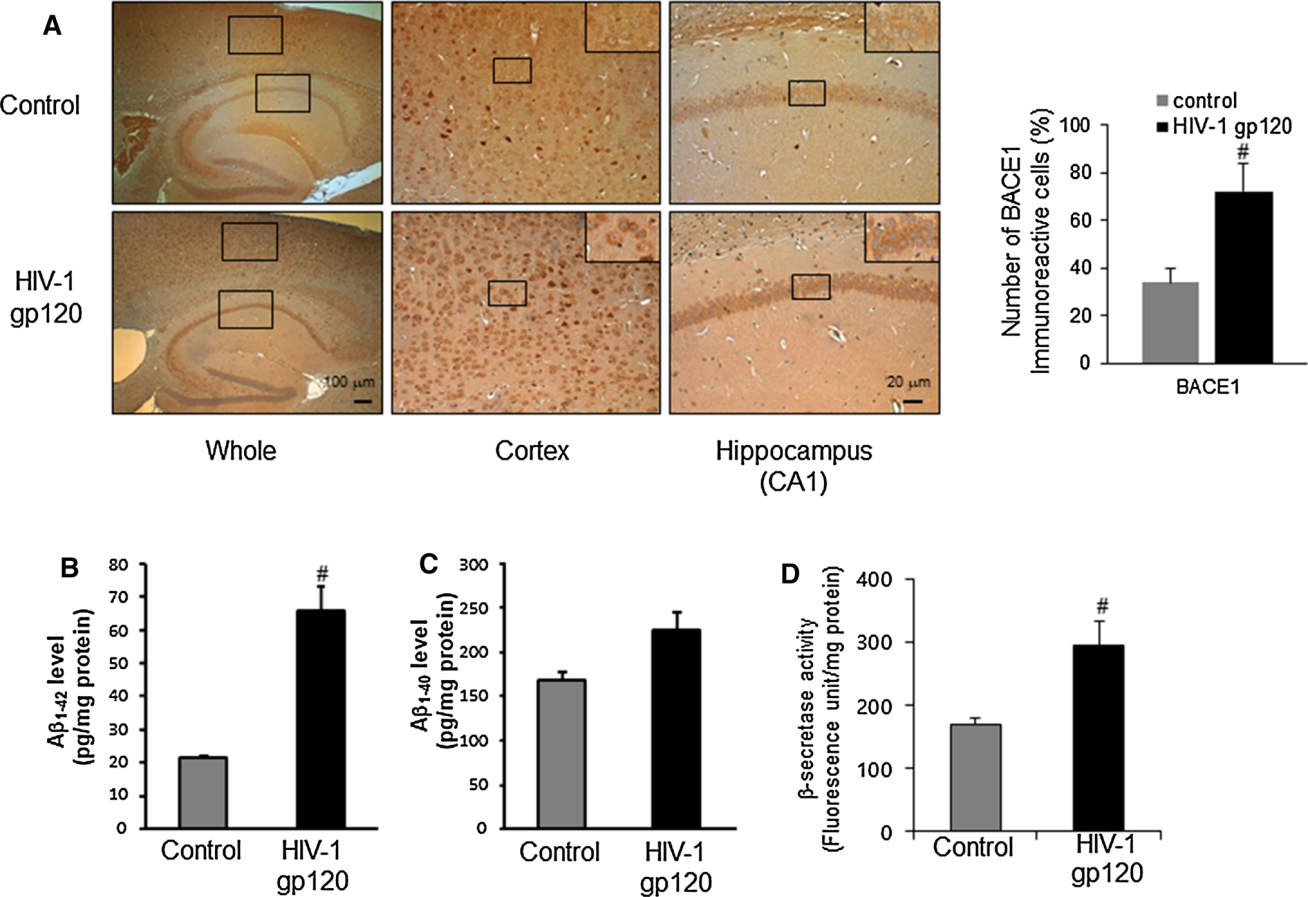
Elevation effects of amyloidogenic proteins on HIV-1 gp120-injected mice brain. **A** Immunoreactive cells of anti-BACE1 antibody were investigated in the cortex and hippocampus. The present figure is representative for three different experiments with different animal brains. The levels of Aβ_1–42_ (**B**) and Aβ_1–40_ (**C**) was assessed by using a specific Aβ_1–42_ ELISA as described in Materials and methods. **D** The activity of β-secretase was investigated by using each assay kit as described in Materials and methods. Values measured from each group of mice were calibrated by amount of protein and expressed as mean ± SD (n = 5). #, Significantly different from control group (*p* < 0.05). Control, saline-injected group. HIV-1 gp120, HIV-1 gp120-injected group

Many studies have reported histopathological findings such as neuroinflammation and Aβ accumulation in the brain of AIDS patients. To determine whether Aβ accumulation is accompanied by HIV-1 gp120-induced memory dysfunction, we investigated the amyloidogenic effects of HIV-1 gp120-injection. Immunohistochemical analysis using an Aβ_1–42_ specific antibody showed Aβ deposition in the cortex and hippocampus regions of HIV-1 gp120-injected mouse brains but not in saline-injected mouse brains (Fig. 3A). To define the Aβ accumulation in the HIV-1 gp120-injected mouse brain, we performed Congo red staining and found congophilic Aβ plaques in the HIV-1 gp120-injected mouse brains (Fig. 3B). To confirm the presence of each Aβ plaque, we performed immunofluorescence double-staining using an Aβ_1–42_ specific antibody and thioflavin S and we observed a plaque double-stained with both Aβ_1–42_ antibody and thioflavin S (Fig. 3C). Then, we investigated the expression level of amyloidogenesis-related proteins using Western blotting and found elevated expression of full-length APP, C99, Aβ, and BACE1 in the HIV-1 gp120-injected mouse brain (Fig. 3D). Moreover, the expression of neuronal β-secretase (BACE1) and the number of BACE1-positive cells were significantly increased by the HIV-1 gp120-injection (Fig. 4A).

Next, ELISA was performed to quantitatively measure Aβ_1–40_ and Aβ_1–42_ levels in the mouse brains. Both Aβ_1–40_ and Aβ_1–42_ levels in the HIV-1 gp120-injected mouse brains were significantly higher compared with the levels of saline-injected mouse brains. The Aβ_1–42_ protein level was 21.44 ± 0.98 pg/mg in the control group brains and 65.51 ± 7.83 pg/mg in the HIV-1 gp120-injected mouse brains (Fig. 4B and C). To determine how amyloid production was elevated by HIV-1 gp120-injection, we analyzed β-secretase activity in the brain and found it to be significantly increased with following the administration of the HIV-1 gp120-injection (Fig. 4D).

### HIV-1 gp120 induced the elevation of iNOS and COX-2 in mouse brain

**Fig. 5:**
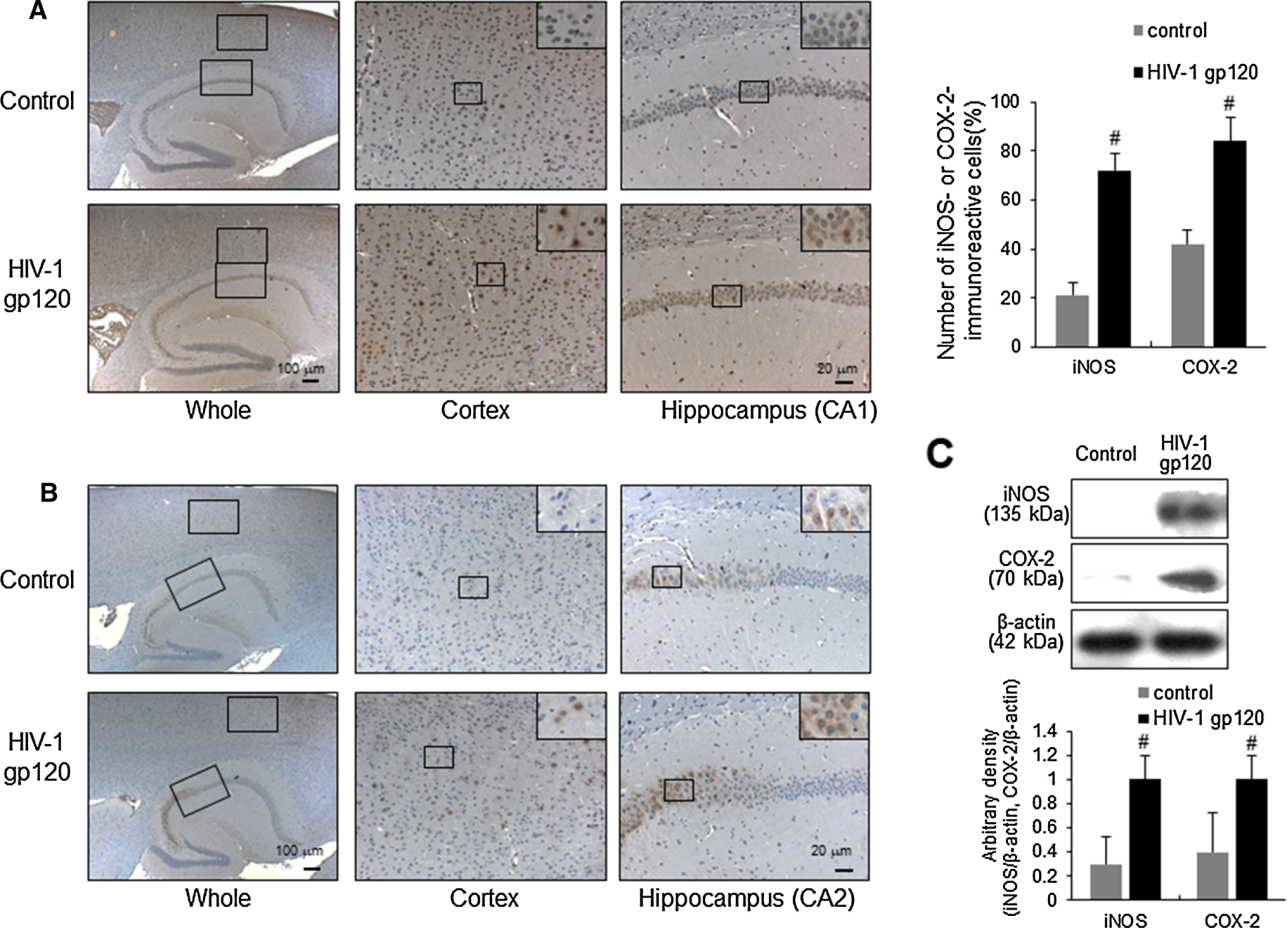
Elevated effects of neuroinflammatory proteins on HIV-1 gp120-injected mice brain. Immunoreactive cells of iNOS (**A**) or COX-2 (**B**) antibody were detected in the cortex and hippocampus. 6 μm-thick sections of brains from mice were incubated with anti-iNOS or anti-COX-2 antibodies, and then the biotinylated secondary antibody. It was counterstained by hematoxylin. The resulting tissue was viewed with a microscope. The present figure is representative for three different experiments with different animal brains. **C** The expression of iNOS and COX-2 were detected by western blotting using specific antibodies. β-Actin protein was used here as an internal control. #, Significantly different from control group (*p* < 0.05). Control, saline-injected group. HIV-1 gp120, HIV-1 gp120-injected group

To investigate if the HIV-1 gp120-induced memory impairment and Aβ accumulation were induced via neuroinflammation, the expression of iNOS was determined by immunohistochemical analysis. Upon HIV-1 gp120 treatment, the number of brown-colored (iNOS-labeled) cells was significantly higher in both the cerebral cortex and hippocampus of gp120-injected mice than in those of the control mice (Fig. 5A). Parallel with the expression level of iNOS detected by immunohistochemical analysis, Western blot analysis also showed that iNOS expression was significantly increased by gp120 injection into the mouse brain (Fig. 5C). Because NO can induce COX-2 expression, and COX-2 is an enzyme that regulates inflammation, we investigated the expression of COX-2 through immunohistochemical and Western blot analysis, which showed that HIV-1 gp120 injection also increased COX-2 expression (Fig. 5B and C).

### HIV-1 gp120 induces the activation of astrocytes and microglia in mouse brain

**Fig. 6:**
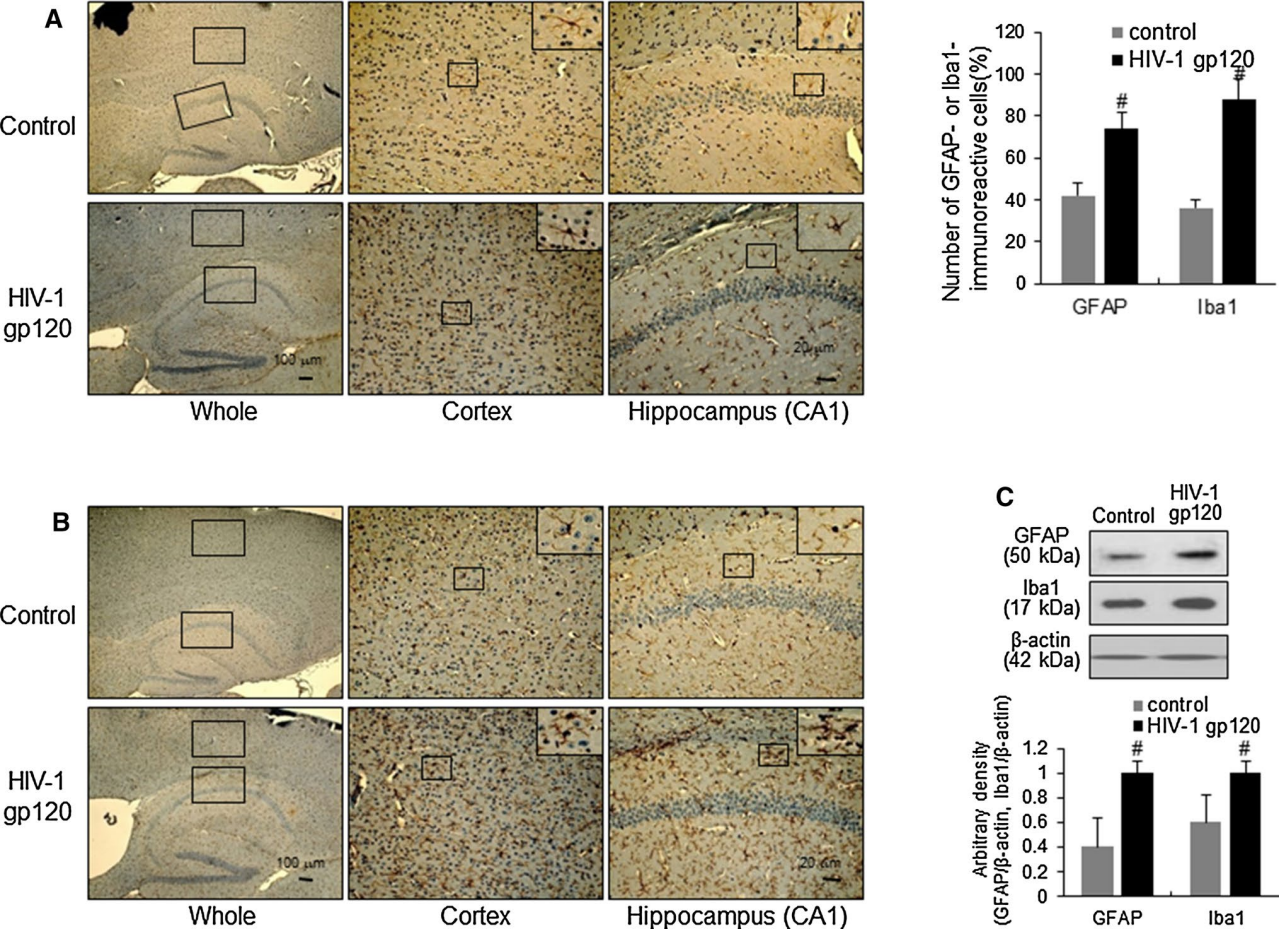
Elevated effect of neuroinflammation on HIV-1 gp120-injected mice brain. **A** Immunoreactive cells of anti-GFAP antibody were investigated in the cortex and hippocampus. **B** Immunoreactive cells of anti-Iba1 antibody were investigated in the cortex and hippocampus. The present figure is representative for three different experiments with different animal brains. **C** The level of GFAP and Iba1 was detected by western blotting using specific antibodies in mice brain. β-Actin protein was used as an internal control. #, Significantly different from control group (*p* < 0.05). Control, saline-injected group. HIV-1 gp120, HIV-1 gp120-injected group

Activation of neuroglia has also been implicated in amyloidogenesis and neuronal cell death during the development of AD. To investigate how HIV-1 gp120 injection induces the activation of astrocytes and microglia, we performed immunohistochemical analysis of GFAP- and Iba1-reactive cells in the brain GFAP- and Iba1-reactive cells were significantly more numerous in the cerebral cortex and hippocampus of gp120-injected mice than those in control mice (Fig. 6A and B). Paralleled with the immunohistochemical results, Western blot analysis also showed that GFAP and Iba1 levels were increased in the brains of gp120-injected mice (Fig. 6C).

### HIV-1 gp120 induced neuroinflammation

**Fig. 7:**
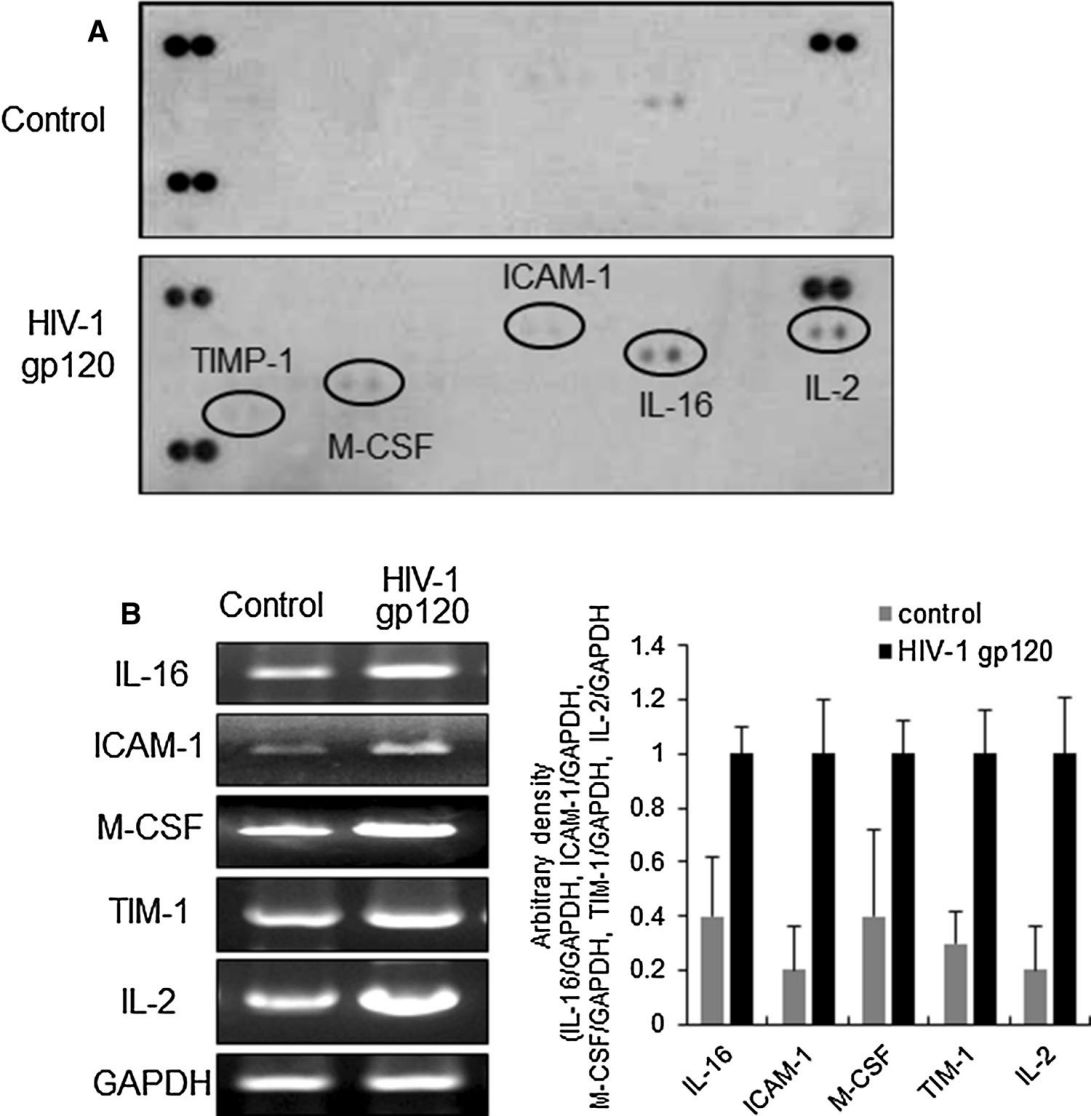
Effect of neuroinflammation on HIV-1 gp120-injected mice brain. **A** Cytokine profiles of saline- or gp120-injected brain. **B** The level of IL-16, ICAM-1, M-CSF, TIM-1, IL-2 and GAPDH expression as described in Materials and methods. Control, saline-injected group. HIV-1 gp120, HIV-1 gp120-injected group

To ascertain the cause of memory impairment induced by HIV-1 gp120 injection, we investigated several cytokine levels. Protein immuno-array results showed increased levels of IL-6, ICAM-1, M-CSF, TIM, and IL-2 (Fig. 7A). Moreover, RT-PCR analysis confirmed that HIV-1 gp120 injection increased the expression of these neuroinflammatory cytokines in the brain (Fig. 7B).

## Discussion

The most important finding in this study was that administration of the HIV-1 gp120 protein induced memory impairment as well as amyloidogenesis and neuroinflammation in the mouse brain. It has been reported that HIV-1 proteins, including env, can directly influence the CNS and activate neuroinflammatory pathways, leading to neuronal injury and dysfunction. In addition, abnormal Aβ, a pathological hallmark of AD, was found to be deposited in individuals suffering from HIV-1 infection (Anderson et al. 2002; Zhang et al. 2015).

HIV-associated neurocognitive disorder (HAND) is a common primary neurological disorder associated with HIV infection. It is known that HAND patients often develop cognitive impairment, motor dysfunction and speech problems. Clinical severity of HAND ranges from asymptomatic neurocognitive impairment and mild neurocognitive disorder to HIV-associated dementia (HAD) (Ru and Tang 2017). Our study showed that injection of HIV-1 gp120 protein in mouse brain induced learning and memory impairment through water maze, probe and step-through tests. The impairment on the learning and memory capability is suggested to be caused by neuropathological change in HIV-1 gp120 injection.

Viral proteins take part in the inflammatory processes of neuronal cells and the activation of glial cells (González-Scarano and Martín-García 2005; Peng et al. 2011; F Hauser et al. 2012). Microglial cells play a fundamental role in the pathogenesis of neurodegenerative diseases such as AD. HIV-1 infection causes glial cells to release several factors, including inflammatory factors that aggravate astrocytes and neurons. The activation of astrocytes reportedly leads to neuronal death and exhibits a correlation with brain damage associated with HIV-1 infection through sustained neuroinflammation (Mollace et al. 2001; Anderson et al. 2002; Minghetti et al. 2004). Positron emission tomography results for AIDS patients also demonstrated microglial activation (Zhang et al. 2012; Garvey et al. 2014). In addition, enhanced glial expression was observed among people with HIV-associated neurodegenerative disease. Therefore, excessive glial activation and neuroinflammation can contribute to cognitive impairment (Coughlin et al. 2014). Furthermore, one of two cleavages of HIV-1 Env protein, the gp120 protein has been demonstrated to release TNF-α and IL-1β, which elicited neuronal apoptosis in numerous ex vivo and in vivo studies (Garden et al. 2002; Bachis et al. 2009). Similar to this effect, other cytokines such as IL-2, IL-16, TIM-1, M-CSF, and ICAM-1 were elevated by HIV-1 gp120 protein in this study. Regarding these cytokines, there is a reported that association between polymorphisms in IL-16 and IL-2 genes and the risk of late-onset Alzheimer’s disease (Anvar et al. 2015; Alves et al. 2016). M-CSF and ICAM were suggested as biomarkers for AD (Laske et al. 2010; Spangenberg et al. 2019). Thus, our animal study demonstrating induction of neuroinflammation by HIV-1 gp120 protein, as evidenced by the enhanced activation of microglial cells and astrocytes, increased COX-2 and iNOS expression, and the release of the several cytokines, supports the clinical findings regarding the correlation between HIV-1 infection and AD.

Activation of microglial and astrocyte cells and the onset of inflammatory reactions and related immunological responses also contribute to Aβ accumulation. Recombinant gp120-injected primary hippocampal cells demonstrated the promotion of Aβ_1–42_ secretion (Aksenov et al. 2010). Increased expression of BACE1, an enzyme involved in the formation of Aβ, was reported in HIV^+^ patients (Stern et al. 2018). It also was found that Aβ is accumulated in HIV-1-infected individuals, even though the site of Aβ deposition showed a discrepancy in the AD brain (Achim et al. 2009; Andras and Toborek 2013). In the present study, we found that administration of the HIV-1 gp120 protein enhanced BACE expression, β-secretase activity, and Aβ accumulation, thus accelerating memory dysfunction (Lee et al. 2010). It is not yet clear how HIV-1 gp120 protein increases Aβ production; however, it is noteworthy that HIV-1 promotes Aβ production through activated β- and γ-secretase activities (Kim et al. 2013; Chai et al. 2017), and these effects may be associated with the modification of lipid rafts by extracellular vesicles by the virus (Mukhamedova et al. 2020). Several viruses, such as cytomegalovirus, herpes simplex virus, HIV-1, severe acute respiratory syndrome (SARS) coronavirus, and SARS-CoV-2, have been found to be related to the cause of neurodegenerative diseases, such as AD (Lurain et al. 2013; Mawanda and Wallace 2013; Barnes et al. 2015; Ardura-Fabregat et al. 2017; Harris and Harris 2018; Lewczuk et al. 2018; Verkhratsky et al. 2019; Devanand et al. 2020; Hampel et al. 2020; Uddin et al. 2020), and clinical associations among viral infections and β-amyloid accumulation and neuroinflammation have also been observed. Therefore, the repurposing of anti-viral drugs as AD-treatment drugs could be possible. This present study demonstrated that viral infection could affect AD development and there may be an association between the amyloidogenic and neuroinflammatory properties of viruses.

## References

[CR1] Achim CL, Adame A, Dumaop W, Everall IP, Masliah E, Neurobehavioral Research C (2009). Increased accumulation of intraneuronal amyloid beta in HIV-infected patients. J Neuroimmune Pharmacol.

[CR2] Aksenov M, Aksenova M, Mactutus C, Booze R (2010). HIV-1 protein-mediated amyloidogenesis in rat hippocampal cell cultures. Neurosci Lett.

[CR3] Alves S, Churlaud G, Audrain M, Michaelsen-Preusse K, Fol R, Souchet B, Braudeau J, Korte M, Klatzmann D, Cartier N (2016). Interleukin-2 improves amyloid pathology, synaptic failure and memory in Alzheimer’s disease mice. Brain.

[CR4] Anderson E, Zink W, Xiong H, Gendelman HE (2002). HIV-1-associated dementia: a metabolic encephalopathy perpetrated by virus-infected and immune-competent mononuclear phagocytes. J Acquir Immune Defic Syndr.

[CR5] Andras IE, Toborek M (2013). Amyloid beta accumulation in HIV-1-infected brain: the role of the blood brain barrier. IUBMB Life.

[CR6] Anvar NE, Saliminejad K, Ohadi M, Kamali K, Daneshmand P, Khorshid HR (2015). Association between polymorphisms in Interleukin-16 gene and risk of late-onset Alzheimer’s disease. J Neurol Sci.

[CR7] Ardura-Fabregat A, Boddeke E, Boza-Serrano A, Brioschi S, Castro-Gomez S, Ceyzeriat K, Dansokho C, Dierkes T, Gelders G, Heneka MT, Hoeijmakers L, Hoffmann A, Iaccarino L, Jahnert S, Kuhbandner K, Landreth G, Lonnemann N, Loschmann PA, Mcmanus RM, Paulus A, Reemst K, Sanchez-Caro JM, Tiberi A, Van Der Perren A, Vautheny A, Venegas C, Webers A, Weydt P, Wijasa TS, Xiang X, Yang Y (2017). Targeting neuroinflammation to treat Alzheimer’s disease. CNS Drugs.

[CR8] Bachis A, Biggio F, Major EO, Mocchetti I (2009). M-and T-tropic HIVs promote apoptosis in rat neurons. J Neuroimmune Pharmacol.

[CR9] Barnes LL, Capuano AW, Aiello AE, Turner AD, Yolken RH, Torrey EF, Bennett DA (2015). Cytomegalovirus infection and risk of Alzheimer disease in older black and white individuals. J Infect Dis.

[CR10] Blennow K, De Leon MJ, Zetterberg H (2006). Alzheimer’s disease. Lancet.

[CR11] Chai Q, Jovasevic V, Malikov V, Sabo Y, Morham S, Walsh D, Naghavi MH (2017). HIV-1 counteracts an innate restriction by amyloid precursor protein resulting in neurodegeneration. Nat Commun.

[CR12] Choi DY, Lee YJ, Lee SY, Lee YM, Lee HH, Choi IS, Oh KW, Han SB, Nam SY, Hong JT (2012). Attenuation of scopolamine-induced cognitive dysfunction by obovatol. Arch Pharm Res.

[CR13] Coughlin JM, Wang Y, Ma S, Yue C, Kim PK, Adams AV, Roosa HV, Gage KL, Stathis M, Rais R (2014). Regional brain distribution of translocator protein using [11 C] DPA-713 PET in individuals infected with HIV. J Neurovirol.

[CR14] Devanand DP, Andrews H, Kreisl WC, Razlighi Q, Gershon A, Stern Y, Mintz A, Wisniewski T, Acosta E, Pollina J, Katsikoumbas M, Bell KL, Pelton GH, Deliyannides D, Prasad KM, Huey ED (2020). Antiviral therapy: Valacyclovir Treatment of Alzheimer’s Disease (VALAD) Trial: protocol for a randomised, double-blind, placebo-controlled, treatment trial. BMJ Open.

[CR15] Ferri CP, Sousa R, Albanese E, Ribeiro WS, Honyashiki M (2009) World Alzheimer Report 2009—executive summary. In: Prince M, Jackson J (eds) Alzheimer’s disease international, London, pp 1–22. 10.18356/1d3bc170-en

[CR16] Garden GA, Budd SL, Tsai E, Hanson L, Kaul M, D’emilia DM, Friedlander RM, Yuan J, Masliah E, Lipton SA (2002). Caspase cascades in human immunodeficiency virus-associated neurodegeneration. J Neurosci.

[CR17] Garvey LJ, Pavese N, Politis M, Ramlackhansingh A, Brooks DJ, Taylor-Robinson SD, Winston A (2014). Increased microglia activation in neurologically asymptomatic HIV-infected patients receiving effective ART. Aids.

[CR18] González-Scarano F, Martín-García J (2005). The neuropathogenesis of AIDS. Nat Rev Immunol.

[CR19] Hampel H, Caraci F, Cuello AC, Caruso G, Nistico R, Corbo M, Baldacci F, Toschi N, Garaci F, Chiesa PA, Verdooner SR, Akman-Anderson L, Hernandez F, Avila J, Emanuele E, Valenzuela PL, Lucia A, Watling M, Imbimbo BP, Vergallo A, Lista S (2020). A path toward precision medicine for neuroinflammatory mechanisms in Alzheimer’s disease. Front Immunol.

[CR20] Harris SA, Harris EA (2018). Molecular mechanisms for herpes simplex virus type 1 pathogenesis in Alzheimer’s disease. Front Aging Neurosci.

[CR21] Hauser FK, Fitting S, Dever MS, Podhaizer ME, Knapp EP (2012). Opiate drug use and the pathophysiology of neuroAIDS. Curr HIV Res.

[CR22] Kamat A, Lyons JL, Misra V, Uno H, Morgello S, Singer EJ, Gabuzda D (2012). Monocyte activation markers in cerebrospinal fluid associated with impaired neurocognitive testing in advanced HIV infection. J Acquir Immune Defic Syndr.

[CR23] Kaul M, Garden GA, Lipton SA (2001). Pathways to neuronal injury and apoptosis in HIV-associated dementia. Nature.

[CR24] Kim J, Yoon JH, Kim YS (2013). HIV-1 Tat interacts with and regulates the localization and processing of amyloid precursor protein. PLoS ONE.

[CR25] Laske C, Stransky E, Hoffmann N, Maetzler W, Straten G, Eschweiler GW, Leyhe T (2010). Macrophage colony-stimulating factor (M-CSF) in plasma and CSF of patients with mild cognitive impairment and Alzheimer’s disease. Curr Alzheimer Res.

[CR26] Lee YJ, Han SB, Nam SY, Oh KW, Hong JT (2010). Inflammation and Alzheimer’s disease. Arch Pharm Res.

[CR27] Lewczuk P, Riederer P, O’bryant SE, Verbeek MM, Dubois B, Visser PJ, Jellinger KA, Engelborghs S, Ramirez A, Parnetti L, Jack CR, Teunissen CE, Hampel H, Lleo A, Jessen F, Glodzik L, De Leon MJ, Fagan AM, Molinuevo JL, Jansen WJ, Winblad B, Shaw LM, Andreasson U, Otto M, Mollenhauer B, Wiltfang J, Turner MR, Zerr I, Handels R, Thompson AG, Johansson G, Ermann N, Trojanowski JQ, Karaca I, Wagner H, Oeckl P, Van Waalwijk Van Doorn L, Bjerke M, Kapogiannis D, Kuiperij HB, Farotti L, Li Y, Gordon BA, Epelbaum S, Vos SJB, Klijn CJM, Van Nostrand WE, Minguillon C, Schmitz M, Gallo C, Lopez Mato A, Thibaut F, Lista S, Alcolea D, Zetterberg H, Blennow K, Kornhuber J, Members of the Wfsbp Task Force Working on This Topic: Peter Riederer CGDKaLMFT (2018). Cerebrospinal fluid and blood biomarkers for neurodegenerative dementias: an update of the Consensus of the Task Force on Biological Markers in Psychiatry of the World Federation of Societies of Biological Psychiatry. World J Biol Psychiatry.

[CR28] Lurain NS, Hanson BA, Martinson J, Leurgans SE, Landay AL, Bennett DA, Schneider JA (2013). Virological and immunological characteristics of human cytomegalovirus infection associated with Alzheimer disease. J Infect Dis.

[CR29] Mawanda F, Wallace R (2013). Can infections cause Alzheimer’s disease?. Epidemiol Rev.

[CR30] Minghetti L, Visentin S, Patrizio M, Franchini L, Ajmone-Cat MA, Levi G (2004). Multiple actions of the human immunodeficiency virus type-1 Tat protein on microglial cell functions. Neurochem Res.

[CR31] Mollace V, Nottet HS, Clayette P, Turco MC, Muscoli C, Salvemini D, Perno CF (2001). Oxidative stress and neuroAIDS: triggers, modulators and novel antioxidants. Trends Neurosci.

[CR32] Morris R (1984). Developments of a water-maze procedure for studying spatial learning in the rat. J Neurosci Methods.

[CR33] Mukhamedova N, Huynh K, Low H, Meikle PJ, Sviridov D (2020). Isolation of lipid rafts from cultured mammalian cells and their lipidomics analysis. Bio Protoc.

[CR34] Ortega M, Ances BM (2014). Role of HIV in amyloid metabolism. J Neuroimmune Pharmacol.

[CR35] Peng H, Sun L, Jia B, Lan X, Zhu B, Wu Y, Zheng J (2011). HIV-1-infected and immune-activated macrophages induce astrocytic differentiation of human cortical neural progenitor cells via the STAT3 pathway. PLoS ONE.

[CR36] Ru W, Tang S-J (2017). HIV-associated synaptic degeneration. Mol Brain.

[CR37] Shadfar S, Hwang CJ, Lim MS, Choi DY, Hong JT (2015). Involvement of inflammation in Alzheimer’s disease pathogenesis and therapeutic potential of anti-inflammatory agents. Arch Pharm Res.

[CR38] Shah A, Verma AS, Patel KH, Noel R, Rivera-Amill V, Silverstein PS, Chaudhary S, Bhat HK, Stamatatos L, Singh DP (2011). HIV-1 gp120 induces expression of IL-6 through a nuclear factor-kappa B-dependent mechanism: suppression by gp120 specific small interfering RNA. PLoS ONE.

[CR39] Spangenberg E, Severson PL, Hohsfield LA, Crapser J, Zhang J, Burton EA, Zhang Y, Spevak W, Lin J, Phan NY, Habets G, Rymar A, Tsang G, Walters J, Nespi M, Singh P, Broome S, Ibrahim P, Zhang C, Bollag G, West BL, Green KN (2019). Sustained microglial depletion with CSF1R inhibitor impairs parenchymal plaque development in an Alzheimer’s disease model. Nat Commun.

[CR40] Stern AL, Ghura S, Gannon PJ, Akay-Espinoza C, Phan JM, Yee AC, Vassar R, Gelman BB, Kolson DL, Jordan-Sciutto KL (2018). BACE1 mediates HIV-associated and excitotoxic neuronal damage through an APP-dependent mechanism. J Neurosci.

[CR41] Uddin MS, Kabir MT, Rahman MS, Behl T, Jeandet P, Ashraf GM, Najda A, Bin-Jumah MN, El-Seedi HR, Abdel-Daim MM (2020). Revisiting the amyloid cascade hypothesis: from anti-abeta therapeutics to auspicious new ways for Alzheimer’s disease. Int J Mol Sci.

[CR42] Verkhratsky A, Parpura V, Rodriguez-Arellano JJ, Zorec R (2019). Astroglia in Alzheimer’s disease. Adv Exp Med Biol.

[CR43] Zhang Y, Wang M, Li H, Zhang H, Shi Y, Wei F, Liu D, Liu K, Chen D (2012). Accumulation of nuclear and mitochondrial DNA damage in the frontal cortex cells of patients with HIV-associated neurocognitive disorders. Brain Res.

[CR44] Zhang Y, Ouyang Y, Liu L, Chen D (2015) Blood-brain barrier and neuro-AIDS. Eur Rev Med Pharmacol Sci 19:4927–4939. https://www.europeanreview.org/wp/wp-content/uploads/4927-4939.pdf 26744885

